# The Increase in Corneal Stiffness After Accelerated Corneal Cross-Linking in Progressive Keratoconus Using Different Methods of Epithelial Debridement

**DOI:** 10.1167/tvst.13.10.38

**Published:** 2024-10-29

**Authors:** Robert Herber, Dierk Wittig, Felix Lochmann, Lutz E. Pillunat, Frederik Raiskup

**Affiliations:** 1 Faculty of Medicine Carl Gustav Carus, Department of Ophthalmology, TU Dresden, Dresden, Germany; 2 Department of Ophthalmology, University Hospital Carl Gustav Carus, TU Dresden, Germany

**Keywords:** corneal cross-linking (CXL), laser ablation, keratoconus (KC), corneal biomechanics, high order aberrations

## Abstract

**Purpose:**

The purpose of this study was to investigate corneal stiffening after epithelium-off accelerated corneal cross-linking (CXL; 9 mW/cm²) in progressive keratoconus (KC) with different methods of epithelial debridement.

**Methods:**

This was a retrospective, interventional, and non-randomized study. In group 1, the epithelium was removed using a hockey knife (*N* = 45). In group 2 (*N* = 39) and group 3 (*N* = 22), the epithelial thickness was measured by optical coherence tomography (OCT) and the epithelium was ablated by excimer laser, but, in group 3, stromal ablation was performed additionally to correct high order aberrations (HOAs). Corneal biomechanics (integrated invers radius [IIR], stress-strain index [SSI]) and corneal tomography (thinnest corneal thickness [TCT]) were assessed with Corvis ST and Pentacam prior to and 1 month after CXL.

**Results:**

Corneal tomography did not differ among the groups preoperatively (*P* > 0.05). TCT decreased significantly in all groups after surgery (all *P* < 0.05). Nonetheless, corneal biomechanical stiffening was found in all three groups indicated by a decreased IIR and an increased SSI (all *P* < 0.05). For group 3, the HOA improved significantly (*P* < 0.001). Among the groups, there were no significant differences in changes of biomechanical parameters, but TCT was significantly reduced after laser ablation.

**Conclusions:**

Corneal stiffening after CXL is independent from epithelial removal. In particular, despite the removal of stromal tissue to correct HOA, a stiffening effect was achieved in keratoconic corneas, even it was less pronounced compared to mechanical epithelial removal. The reduction in HOA indicates a possible improvement in visual acuity.

**Translation Relevance:**

Cross-linking stiffens the keratoconus independent of epithelial debridement technique and may compensate minor stromal laser ablation.

## Introduction

Corneal biomechanics and corneal ectasia, especially keratoconus (KC), are closely linked. In previous ex vivo studies using stress-strain measurements, corneas affected by KC exhibited reduced biomechanical behavior, as evidenced by lower stress and modulus of elasticity.^1^^,^^2^ KC is a progressive disease with increasing steepening of the corneal curvature, decreasing corneal thickness, and changes in the distribution of corneal epithelial thickness,^3^ with thinning of the epithelium that occurs over the cone, which is surrounded by a thicker epithelial ring. Corneal cross-linking (CXL) has become the gold standard for the treatment of KC, which aims to increase the strength of the cornea through a photo-oxidative process using riboflavin and ultraviolet light type A.^4^^–^^6^ In addition to numerous experimental studies,^7^ these effects have also been demonstrated in vivo using Scheimpflug based air-puff tonometry, with the so-called dynamic corneal response (DCR) parameters (Corvis ST; Oculus Optikgeraete GmbH, Wetzlar, Germany) proving sensitive enough to detect such biomechanical changes after CXL, especially 1 month after treatment.^8^^–^^11^ A recent study confirmed the long-term efficacy of the treatment over a 15-year period.^12^ In addition, a randomized controlled trial confirmed the clinical need that led to US Food and Drug Administration (FDA) approval.^13^ This study also showed that only 25% of treated patients had a significant improvement in visual acuity (gain of more than 2 lines). Consequently, there are a number of patients who benefit from treatment in terms of corneal stability but not visual acuity. Therefore, there is a need for improvement in the visual acuity of patients with KC, especially if they are intolerant to rigid gas permeable or scleral lenses. Kanellopoulos and Asimellis were the first who showed that combining CXL with excimer laser ablation (“Athens protocol”) can improve vision, with laser ablation being performed first and CXL immediately afterwards.^14^ They showed a significant increase in both best corrected and uncorrected visual acuity.^14^^–^^16^ However, this study also reported a reduction in corneal tissue of 80 µm. To overcome this potential problem, Gore et al. published a protocol aiming to correct only ocular wavefront aberrations using a transepithelial photorefractive keratectomy (tPRK) combined with immediate CXL, without the primary goal to reduce the sphere and cylinder of refraction.^17^ The protocol saves tissue and could be applied to mild and moderate KC with good visual results. However, the removal of tissue and thus the reduction of corneal thickness by laser correction stands in contradiction with the disease itself, as KC has a biomechanically weakened cornea. The aim of this study was to demonstrate in vivo that the combination of corneal wavefront-guided tPRK with CXL is not inferior to epithelium-off (epi-off) accelerated CXL in terms of biomechanical outcome 1 month after the treatment.

## Methods

This was a retrospective and monocentric study conducted in a university hospital setting. The study protocol (NCT04251143) was approved by the ethics committee in accordance with the Declaration of Helsinki. Patients had to sign an informed consent form to be included in the study. Only patients with KC with confirmed progression in corneal tomography and who received CXL treatment between 2017 and 2023 were included. The progression criterion was an increase in maximum keratometry (K max) of more than 1 diopter (D) based on the definition of the national healthcare system in Germany.^18^ Patients received corneal tomography (Pentacam; Oculus Optikgeraete GmbH, Wetzlar, Germany) and biomechanical (Corvis ST; Oculus Optikgeraete GmbH, Wetzlar, Germany) measurement, pre- and postoperatively. Exclusion criteria were previous corneal surgeries, such as CXL, laser vision correction treatments, or keratoplasty, as well as pregnancy. Only one eye per patient was included in this study and consecutively assigned to one of the three groups. Wearing of contact lenses was discontinued for 14 days prior to each examination.

### Surgical Procedure

The surgical preparations were carried out exactly as described in previous studies.^19^ In group 1, the epithelium was removed during the procedure using a hockey knife (mechanical epi-off CXL). In the second group, the epithelium was measured using an anterior segment (swept-source) optical coherence tomograph (AS-OCT; ANTERION, Heidelberg Engineering, Heidelberg, Germany). The mean epithelium thickness (ET) of a central zone of 3 mm was determined and entered into the laser planning software (SCHWIND CAM, Schwind eye-tec solutions, Kleinostheim, Germany). The ablation depth of the tPTK-asst.-epi-off CXL was set to *ET +* 5 µm with a diameter of 8 mm. For group 3, corneal wavefront aberrations were measured by a placido-disk based topographer (Keratron Scout, OPTIKON 2000, Roma, Italy) or an AS-OCT (MS-39, CSO, Firenze, Italy). From these measurements, the corneal wavefront aberrations were calculated by the software and exported to the laser planning software (SCHWIND CAM, SCHWIND eye-tech-solutions GmbH, Kleinostheim, Germany). Within the software, the first step was to adjust the patients’ actual ET. Additionally, the optical zone of the ablation was modified according to the scotopic size of the pupil, however, the zones were between 6.8 and 7.0 mm. The software's algorithm enables the reduction of tissue ablation (minimizing the ablation depth function) by removing unnecessary high order aberrations (HOAs) from the calculation of the ablation pattern. The primary treatment target was the reduction of HOAs, but, in some cases, the manifest refraction was also considered, if the ablation depth did not exceed 50 µm in the cone area using the “PRK” mode of the software. The PRK mode represents the stromal ablation of the treatment pattern. For groups 2 and 3, laser ablation as a transepithelial treatment considering epithelium thickness was performed using the SCHWIND Amaris 750 laser (SCHWIND eye-tech-solutions GmbH, Kleinostheim, Germany). Afterward, riboflavin was applied every 2 minutes for 15 minutes, followed by UV-A light irradiation (UV-X 2000, former IROC Innocross AG, Zug, Switzerland). Postoperatively, soft contact lenses were applied on the eye until re-epithelialization was completed. During the first 6 days, antibiotic eye drops (Floxal EDO; Dr. Mann Pharma, Berlin, Germany), steroids (Softacort; Théa Pharma GmbH, Berlin, Germany), and artificial tears (Thealoz Duo, Théa Pharma GmbH) were prescribed. Additionally, dexamethasone steroid (Dexa EDO; Dr. Mann Pharma) eye drops were taken 3 times a day, after day 4. After the re-epithelialization, medication was continued for 3 weeks with steroids together with artificial tears.

**Figure 1.**
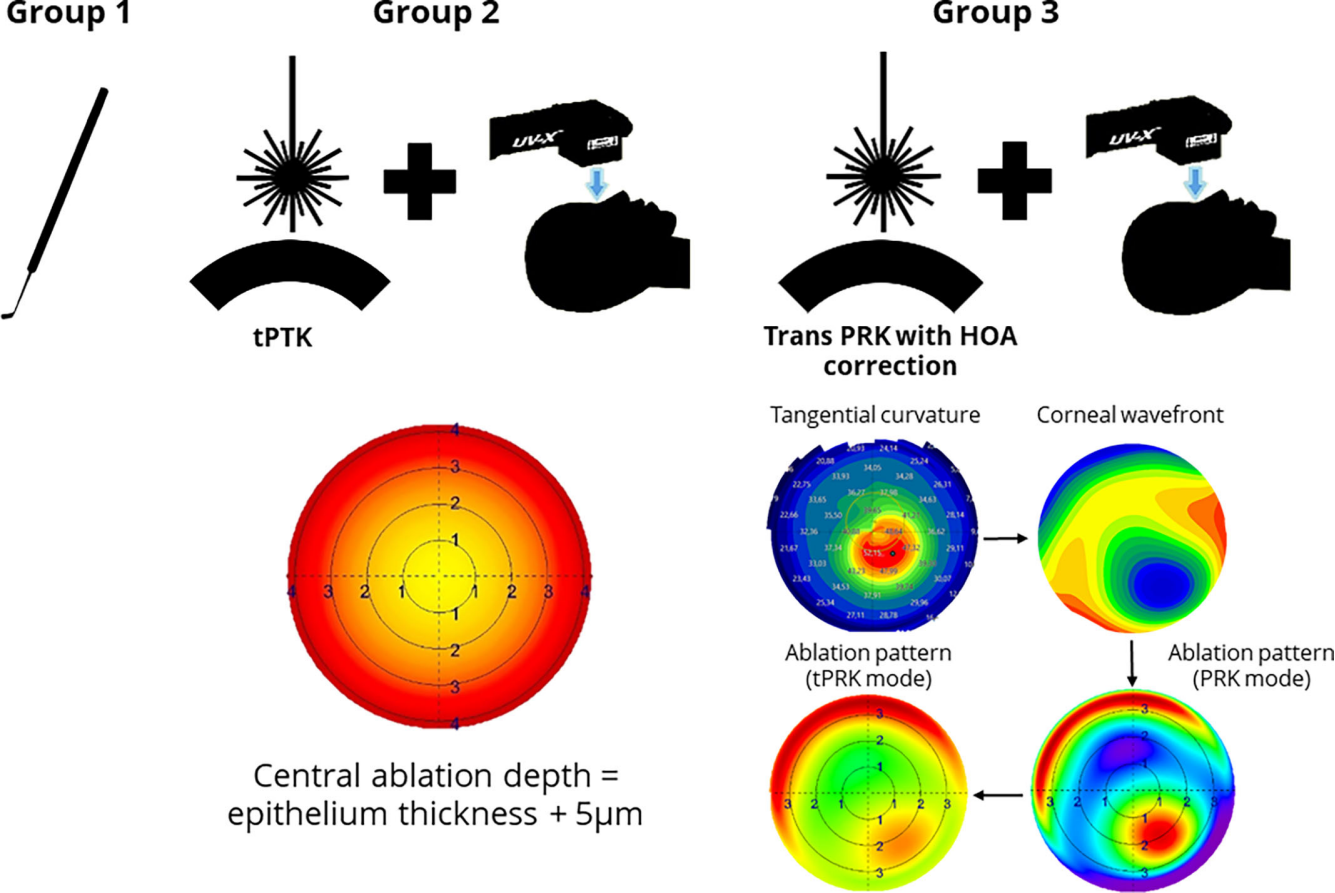
Flowchart of the surgical procedures in each group. *Left*: Utilization of a reusable hockey knife. *Center*: Ablation pattern demonstrated by the central ablation and calculated by epithelium thickness plus 5 µm (*yellow area*). In the periphery, the ablation depth is higher as the laser light cannot enter at a perpendicular angle to the corneal surface. The lights therefore travel a longer way through the epithelium and the epithelium thickness is measured perpendicular to the surface of the cornea. *Right*: From corneal topography or tomography, a corneal wavefront is calculated, then imported into the laser planning software. Ablation pattern is assessed in PRK mode, if ablation depth in the cone (*central red area*) does not exceed 50 µm. A peripheral ablation of more than 50 µm was allowed as corneal thickness is usually thicker in this area.

The CXL protocols are summarized in Table 1 and Figure 1.

**Table 1.** Description of CXL Protocols

Parameter	Mechanical Epi-Off CXL	tPTK-asst. Epi-off CXL	Wf-Guided tPRK CXL
Treatment target	Progressive keratoconus	Progressive keratoconus	Progressive keratoconus
Fluence, total, mJ/cm²	5.4	5.4	5.4
Soak time (interval)	15 min (q2)	15 min (q2)	15 min (q2)
Intensity, mW/cm²	9 mW	9 mW	9 mW
Treatment time	10 min	10 min	10 min
Light source	UV-X 2000	UV-X 2000	UV-X 2000
Irradiation mode	Continuous	Continuous	Continuous
Epithelium status	Off (mechanical abrasion)	Off (transepithelial phototherapeutic keratectomy)	Off (wavefront-guided transepithelial photorefractive keratectomy)
Ablation diameter	8 mm	8 mm	6.8–7.0 mm
Chromophore (centration)	Riboflavin (0.1%)	Riboflavin (0.1%)	Riboflavin (0.1%)
Chromophore carrier	Hydroxypropyl methyl cellulose (HPMC)	Hydroxypropyl methyl cellulose (HPMC)	Hydroxypropyl methyl cellulose (HPMC)
Chromophore osmolarity	Iso-osmolar	Iso-osmolar	Iso-osmolar

### Air Puff Tonometry Using Dynamic Scheimpflug Analyzer

The dynamic Scheimpflug analyzer (Corvis ST; CVS, Oculus, Wetzlar, Germany) records the air-puff induced deformation process of the cornea using an ultra-high-speed camera. The parameters, which were calculated from these measurements are known as DCR parameters and have been described previously.^20^^,^^21^ For this study, only the integrated inverse radius (IIR) and stress-strain index (SSI, version 1^22^) were analyzed as both biomechanical parameters have been shown to be sensitive enough to detect corneal stiffening after CXL.^8^^,^^9^ The IIR describes the concave phase of the cornea during the deformation process. “Integrated” means that all inverse radii (1/R) are summed up between the first and second applanations.^20^ The parameter itself represents the overall stiffness as it depends on geometric properties of the cornea. The SSI best describes the material stiffness of the cornea due to its independence from the intraocular pressure and the corneal thickness.^22^ In addition, the biomechanical corrected intraocular pressure (bIOP)^23^ and the pachymetry values (CVS-CT) were gathered.

### Statistical Analysis

The data were collected using Excel 2016 (Microsoft Corp., Redmond, WA, USA) and analyzed using SPSS version 28 (IBM Statistics, Armonk, NY, USA). The normal distribution of the data was assessed by Q-Q plots and Kolmogorov test. Normally distributed data were analyzed using the *t*-test for pre- and postoperative comparisons in each group. Group comparisons were performed with the 1-way analysis of variance (ANOVA). Dichotomous data were analyzed with the χ² test. Continuous parameters are shown as mean ± standard deviation and pre- and postoperative mean differences as mean ± standard deviation (95% confidence interval). Univariate and multivariate regression analysis was performed. In the multivariate regression analysis, the independent parameters were included backward using the Wald method. The sample size calculation was based on a noninferiority study design to demonstrate noninferiority of the Wf-guided tPRK-CXL group with respect to biomechanical outcomes compared to mechanical epi-off-CXL and tPTK-ass. epi-off-CXL, despite corneal tissue ablation of up to 50 µm in the Wf-guided tPRK-CXL group. The noninferiority cutoff (d) was set at d = 0.95 and d = 0.1 with a standard deviation of 1.14 and 0.101, respectively. These values were based on two previous studies. Padmanabhan et al. showed a biomechanical weakening during the KC progression period for the IIR and SSI parameters of 0.95 ± 1.04 mm^−1^ and −0.10 ± 0.06, respectively.^24^ The mean difference values were chosen as d, due to Wf-guided tPRK that has been hypothesized to weaken the cornea. The standard deviation was taken from a previous study that examined the pre- and postoperative differences after CXL.^9^ The calculation resulted in a minimum of 13 (for IIR) and 18 (for SSI) per group (alpha error = 0.05, 1-power = 0.2; Sealed Envelope Ltd. 2012. Power Calculator for continuous outcome noninferiority trial. [Online] Available from: https://www.sealedenvelope.com/power/continuous-noninferior/ [Accessed Wednesday, May 5, 2024]). A *P* value below 0.05 was assumed to be significant.

## Results

The demographic data did not differ among the groups (Table 2), meaning that especially the severity of KC based on maximum keratometry (K max), thinnest corneal thickness (TCT), A-parameter, and B-parameter (of the ABCD grading system of the corneal tomographer) were comparable among the groups (all *P* > 0.05).

**Table 2.** Demographic Data of the Study Groups Expressed as Mean ± Standard Deviation

	Mechanical Epi-Off CXL	tPTK-asst. Epi-off CXL	Wf-Guided tPRK CXL	*P* Value
Number of eyes	45	39	28	—
Laterality	17 (38)/28 (62)	15 (38)/24 (62)	15 (54)/13 (46)	0.355
Right/left eyes (%)				
Gender	31 (69)/14 (31)	23 (59)/16 (41)	21 (75)/7 (25)	0.365
Male/female				
Age, y	29.5 ± 10.1	29.5 ± 10.7	27.0 ± 8.0	0.527
K mean, D	46.4 ± 2.7	46.0 ± 2.9	45.2 ± 1.8	0.205
K max, D	54.1 ± 4.1	53.6 ± 5.3	52.6 ± 3.1	0.360
TCT, µm	472 ± 33	482 ± 31	481 ± 29	0.321
A-parameter	2.1 ± 1.0	2.4 ± 1.4	1.7 ± 0.7	0.088
B-parameter	3.2 ± 1.3	3.8 ± 1.7	2.9 ± 1.2	**0.041** ^a^
C-parameter	1.5 ± 0.8	1.7 ± 0.8	1.7 ± 0.8	0.637

A-parameter, stage of anterior curvature of the ABCD grading system; B-parameter, stage of posterior curvature of the ABCD grading system; C-parameter, stage of thinnest corneal thickness of the ABCD grading system; K mean, mean keratometry value of the central 3 mm zone; K max, maximum keratometry; TCT, thinnest corneal thickness.

Statistical significance (*P* < 0.05) is marked in bold face.

a Pairwise comparison between all three groups and applied Bonferroni correction did not show any statistical significance among the groups.

For group 3, the mean stromal ablation (PRK mode) was 17.6 ± 7.5 µm and 35.2 ± 10.0 µm in the central and cone area, respectively. As transepithelial treatment, the mean laser ablation was 66.7 ± 8.4 µm and 87.3 ± 11.7 µm in the central and cone area estimated by the laser software, respectively. The mean optical treatment zone was 7.0 ± 0.2.

### Biomechanical Assessment Before and After CXL

**Figure 2.**
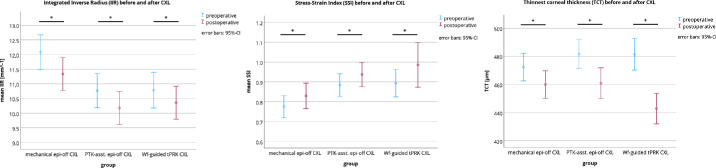
Mean pre- and postoperative values of integrated inverse radius (IIR; *left*), stress strain index (SSI; *center*), and thinnest corneal thickness (TCT; *right*). A *P* value < 0.05 indicated statistical significance.

The pre- and postoperative changes are displayed in Table 3. The IIR decreased statistically significantly by −0.7 ± 0.9 (−1.0 to −0.5), −0.6 ± 1.1 (−0.9 to −0.2), and −0.4 ± 0.7 (−0.7 to −0.1) mm^−1^ for the mechanical epi-off CXL, tPTK-asst. epi-off CXL, and Wf-guided tPRK CXL group (all *P* < 0.05), respectively (Fig. 2). The SSI increased statistically significantly by 0.05 ± 0.12 (0.01–0.09), 0.05 ± 0.13 (0.01–0.09), and 0.09 ± 0.19 (0.02–0.16) for the mechanical epi-off CXL, tPTK-asst. epi-off CXL, and Wf-guided tPRK CXL group (all *P* < 0.05), respectively. It should be noted that the standard deviation of SSI increased postoperatively in the Wf-guided tPRK-CXL group, indicating a greater variance in measurement results after treatment.

**Table 3.** Pre- and Postoperative Comparison of Corvis ST Parameters

Parameter		Mechanical Epi-Off CXL	*P* Value	tPTK-asst. Epi-Off CXL	*P* Value	Wf-Guided tPRK CXL	*P* Value	*P* Value^a^	*P* Value^b^	*P* Value^c^
CVS-CT	Preoperative	484 ± 34	**<0.001**	508 ± 31	**<0.001**	505 ± 30	**<0.001**			
	Postoperative	462 ± 39		484 ± 36		474 ± 24				
	Mean difference	−22 ± 18		−25 ± 21		−31 ± 16		1.0	0.175	0.564
	(95% CI)	(−27 to −17)		(−31 to −18)		(−37 to −24)				
bIOP, mm Hg	Preoperative	13.4 ± 2.4	**<0.001**	13.6 ± 1.8	**<0.001**	13.2 ± 2.0	**<0.001**			
	Postoperative	15.4 ± 3.1		15.4 ± 2.3		16.0 ± 2.4				
	Mean difference	2.03 ± 2.21		1.8 ± 2.2		2.8 ± 2.1		1.0	0.861	0.394
	(95% CI)	(1.4 to 2.7)		(1.1 to 2.5)		(2.0 to 3.6)				
IIR, mm^−1^	Preoperative	12.1 ± 2.0	**<0.001**	10.8 ± 1.8	**<0.001**	10.8 ± 1.6	**0.005**			
	Postoperative	11.3 ± 1.9		10.2 ± 1.7		10.4 ± 1.5				
	Mean difference	−0.7 ± 0.9		−0.6 ± 1.1		−0.4 ± 0.7		1.0	0.476	1.0
	(95% CI)	(−1.0 to −0.5)		(−0.9 to −0.2)		(−0.7 to −0.1)				
SSI	Preoperative	0.78 ± 0.18	**0.005**	0.88 ± 0.17	**0.014**	0.89 ± 0.18	**0.014**			
	Postoperative	0.83 ± 0.21		0.94 ± 0.18		0.99 ± 0.29				
	Mean difference	0.05 ± 0.12		0.05 ± 0.13		0.09 ± 0.19		1.0	0.822	0.820
	(95% CI)	(0.01 to 0.09)		(0.01 to 0.09)		(0.02 to 0.16)				

Data are expressed as mean ± standard deviation and mean differences additionally with 95% confidence intervals (95% CI).

bIOP, biomechanical-corrected intraocular pressure; CVS-CT, corneal thickness measured with Corvis ST; IIR, integrated inverse radius; SSI, stress strain index.

Statistical significance (*P* < 0.05) is marked in bold face.

a *P* value between mechanical epi-off CXL and tPTK-asst. epi-off CXL.

b *P* value between mechanical epi-off CXL and Wf-guided tPRK CXL.

c *P* value between tPTK-asst. epi-off CXL and Wf-guided tPRK CXL.

As expected, CVS-CT decreased statistically significantly 1 month after CXL in all three groups (all *P* < 0.001), however, the mean differences were not statistically significant among the groups (all *P* < 0.05).

### Tomographic Assessment Before and After CXL

As this study primarily investigated biomechanical changes before and after CXL with or without the combination of laser ablation, tomographic alterations were only the secondary outcome (Table 4). After treatment, a significant improvement was found only in the Wf-guided tPRK CXL group for the maximum keratometry (K max), the index of variance (ISV), and the root mean square of anterior HOAs (anterior RMS-HOA, *P* < 0.001).

**Table 4.** Pre- and Postoperative Comparison of Corneal Tomography

Parameter		Mechanical Epi-Off CXL	*P* Value	tPTK-Asst. Epi-off CXL	*P* Value	Wf-Guided tPRK CXL	*P* Value	*P* Value^a^	*P* Value^b^	*P* Value^c^
K max, D	Preoperative	54.1 to 4.1	**<0.001**	53.6 to 5.3	0.619	52.6 to 3.1	**<0.001**			
	Postoperative	55.4 to 4.4		53.7 to 5.0		49.1 to 2.6				
	Mean difference	1.3 to 1.5		0.1 to 1.7		−3.5 to 2.4		**0.012**	**<0.001**	**<0.001**
	(95% CI)	(0.8 to 1.8)		(−0.4 to –0.7)		(−4.5 to −2.6)				
CCT [µm]	Preoperative	482 to 33	**<0.001**	494 to 33	**<0.001**	491 to 31	**<0.001**			
	Postoperative	469 to 34		478 to 33		470 to 27				
	Mean difference	−13 to 11		−16 to 12		−21 to 14		0.513	**<0.001**	**0.038**
	(95% CI)	(−16 to −10)		(−20 to −13)		(−15 to −3)				
TCT [µm]	Preoperative	472 to 33	**<0.001**	482 to 31	**<0.001**	481 to 28.9	**<0.001**			
	Postoperative	460 to 32		461 to 33		442 to 28.0				
	Mean difference	−12 to 11		−21 to 15		−39 to 18		**0.035**	**<0.001**	**<0.001**
	(95% CI)	(−15 to −9)		(−26 to −16)		(−46 to −31)				
ISV	Preoperative	83.3 to 28.4	**<0.001**	79.4 to 35.1	0.620	73.1 to 20.2	**<0.001**			
	Postoperative	90.2 to 28.0		78.5 to 33.1		56.3 to 20.6				
	Mean difference	6.9 to 6.6		−0.90 to 11.2		−16.9 to 14.0		**0.003**	**<0.001**	**<0.001**
	(95% CI)	(4.9 to 8.9)		(−4.5 to 2.7)		(−22.3 to −11.4)				
Anterior	Preoperative	6.6 to 2.6	0.548	8.8 to 4.0	0.084	4.9 to 2.6	**<0.001**			
RMS-HOA	Postoperative	6.9 to 2.3		8.4 to 4.1		2.2 to 1.3				
	Mean difference	0.3 to 1.2		−0.4 to 1.3		−2.7 to 1.7		0.796	**<0.001**	**<0.001**
	(95% CI)	(−0.7 to 1.3)		(−0.8 to 0.05)		(−3.4 to −2.0)				

Data are expressed as mean ± standard deviation and mean differences additionally with 95% confidence intervals (95% CI).

CCT, central corneal thickness; HOA, high order aberrations; ISV, index of surface variance; K max, maximum keratometry; RMS, root mean square; TCT, thinnest corneal thickness.

Statistical significance (*P* < 0.05) is marked in bold face.

a P-value between mechanical epi-off CXL and tPTK-asst. epi-off CXL.

b P-value between mechanical epi-off CXL and Wf-guided tPRK CXL.

c P-value between tPTK-asst. epi-off CXL and Wf-guided tPRK CXL.

Contrarily to the CVS-CT measurement, the central corneal thickness (CCT) and TCT statistically significantly decreased in all groups after the treatment (*P* < 0.001). The changes of CCT were not significant between mechanical epi-off CXL group and the tPTK-asst. epi-off CXL group, whereas the CCT decreased more strongly in the Wf-guided tPRK CXL group in comparison to both of the other groups (*P* < 0.05). Additionally, the decrease in TCT was more pronounced in both laser-assisted CXL groups compared to the mechanical group (*P* < 0.05). As a result, it would be expected that the stiffening effect would be less distinct. Interestingly, when comparing the pre- and postoperative mean changes of biomechanical parameters between the groups, no statistical difference was found for IIR and SSI (all *P* > 0.05; see Table 3), suggesting that a similar stiffening effect of the CXL procedure is produced regardless of the method of epithelial removal. However, this result should be interpreted with caution, as the statistical significance of the pairwise post hoc comparison is low due to the fact that the study design was defined as a noninferiority study.

### Relationship Between Changes in Corneal Thickness Biomechanical Parameters

**Figure 3.**
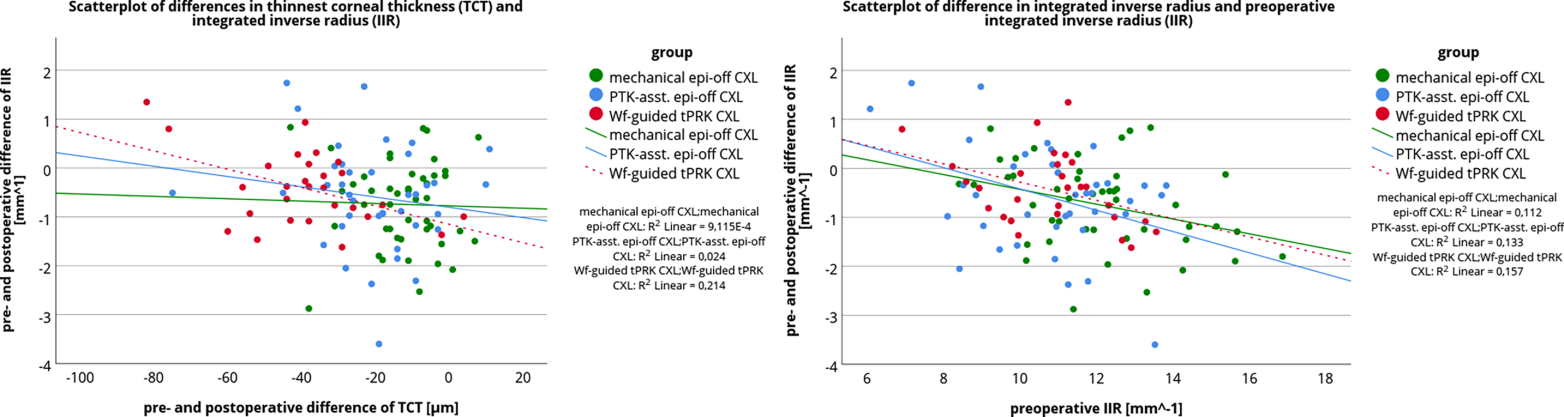
Scatterplot of pre- and postoperative differences in integrated invers radius (IIR) with preoperative and postoperative differences of thinnest corneal thickness (TCT; *left*) and preoperative integrated inverse radius (IIR; *right*). The *lines* show the linear relationship.

**Figure 4.**
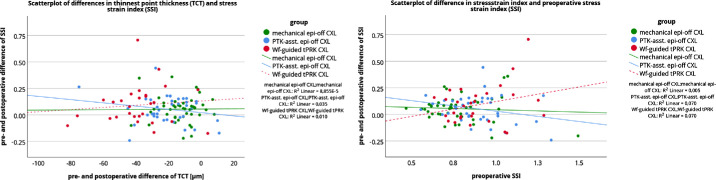
Scatterplot of pre- and postoperative differences in stress strain index (SSI) with preoperative and postoperative differences of thinnest corneal thickness (TCT; *left*) and preoperative stress strain index (SSI; *right*). The lines show the linear relationship.

To support this finding, a multivariate regression analysis was performed. Defining Δ TCT, preoperative value of IIR or SSI, and the factor group (the mechanical Epi-Off-CXL group, the tPTK-ass-Epi-Off-CXL group, and the Wf-guided tPRK-CXL group) as potential influencing factors of the changes in IIR (Δ IIR) and SSI (Δ SSI), the multivariate regression analysis showed a significant effect of preoperative IIR on Δ IIR (*P* < 0.001), but not the group factor or Δ TCT. No relationship was found for Δ SSI. Figure 3 shows the low correlation between Δ TCT and Δ IIR, indicating that the smaller the change in TCT, the greater the decrease in IIR. However, the results should be treated with caution due to the large scatter of the data points. The close relationship between preoperative IIR and Δ IIR is shown in Figure 3. The higher the preoperative IIR was, the greater was the postoperative decrease observed in IIR, in all groups. In addition, the lack of relationship between Δ TCT and Δ SSI as well as between preoperative SSI and Δ SSI is shown in Figure 4.

In addition, the relationship between the central and cone ablation depth with the pre- and postoperative changes in bIOP (Δ bIOP), IIR (Δ IIR), SSI (Δ SSI), and CCT (Δ CCT) or TCT (Δ TCT), was investigated for the Wf-guided tPRK-CXL group (Table 5). In univariate regression analysis, the only significant correlation was found between central ablation depth and Δ CCT (*P* = 0.006), and between cone ablation depth and Δ TCT (*P* = 0.005). Multivariate regression showed the same result for Δ CCT (*P* = 0.003) and Δ TCT (*P* = 0.005). These results suggest that the ablation depth of wavefront-guided tPRK combined with CXL only influences the changes in corneal thickness (CCT and TCT) and not the biomechanical parameters.

**Table 5.** Univariate and Multivariate Regression Analysis to Determine the Relationship of Changes in Biomechanical-Corrected Intraocular Pressure (bIOP), Integrated Inverse Radius (IIR), Stress Strain Index (SSI), Central Corneal Thickness (CCT), and Thinnest Corneal Thickness (TCT) With Central Ablation Depth and Cone Ablation Depth in the Wf-Guided tPRK CXL Group

	Central Ablation Depth				Cone Ablation Depth			
	Univariate Regression		Multivariate Regression		Univariate Regression		Multivariate Regression	
	B	*P* Value	B	*P* Value	B	*P* Value	B	*P* Value
Δ bIOP	−0.666	0.344	—	—	−0.621	0.510	—	—
Δ IIR	−1.415	0.479	−3.352	0.062	1.707	0.522	—	—
Δ SSI	−10.472	0.185	−12.670	0.074	−11.447	0.280	—	—
Δ CCT	−0.279	**0.006**	−0.295	**0.003**	—	—	—	—
Δ TCT	—	—	—	—	−0.283	**0.005**	−0.283	**0.005**

B, beta; bIOP, biomechanical-corrected intraocular pressure; CCT, central corneal thickness, CVS-CT, corneal thickness measured with Corvis ST; Δ, pre- and postoperative difference; IIR, integrated inverse radius; SSI, stress strain index.

Statistical significance (*P* < 0.05) is marked in bold face.

## Discussion

The efficacy of corneal crosslinking as a treatment for keratectasia has been studied extensively. Under ex vivo conditions, the focus has been on corneal stiffening, often demonstrated by stress-strain measurements.^4^^,^^5^^,^^25^ However, examinations of the microstructure of the tissue, for example, collagen diameter, which showed an increase, suggest that CXL mainly occurs in the anterior stroma.^26^^,^^27^ In vivo, topographic and tomographic data of the cornea were used in most studies and showed stability (halting of progression) and a slight flattening of the anterior corneal curvature, indicating a more regularized cornea with partially improved visual acuity.^6^^,^^13^^,^^28^ However, visual improvement after standard or accelerated CXL is not as high as after refractive laser treatment and varies from patient to patient. New CXL protocols have been introduced to solve this problem and improve visual acuity. An appropriate method is the combination of CXL and laser treatment. However, laser treatment itself is contraindicated because keratoconus is a major risk factor for iatrogenic keratectasia after laser vision correction, as the tissue is further weakened by the laser treatment.^29^ Therefore, the aim of this study was to evaluate the biomechanical changes after CXL combined with laser treatment to reduce HOA in progressive keratoconic eyes using the dynamic Scheimpflug analyzer. We chose a 1-month follow-up period to investigate the biomechanical changes in 3 different protocols: the mechanical epi-off CXL, the tPTK-asst. epi-off CXL, and the Wf-guided tPRK CXL.

The main outcome of this study was that the stiffening effect of corneal tissue after CXL was independent from the method of epithelial removal, with the measured biomechanical change being more pronounced in the mechanical epi-off CXL group. This result can be inferred from the changes in IIR, known as a measure of overall stiffness, and SSI, known as a measure of material stiffness. The results can be explained by previous studies that found that CXL stiffens the anterior cornea to a depth of 200 µm,^27^ and this area was equally cross-linked and stiffened in all groups.

The secondary outcome was an observed improvement in anterior surface curvature and a reduction in HOA in the Wf-guided tPRK-CXL group, which could further lead to an enhancement in visual acuity in these patients upon completion of wound healing, a process that typically occurs between 6 and 12 months after treatment.

Recently, the measurement of the stiffening effect of the cornea after CXL in vivo has been made possible by the introduction of new biomechanical parameters in the CVS software. These parameters allow the separate consideration of the inward and outward movement of the cornea during the deformation process induced by the air puff, thus allowing the measurement of corneal elasticity. An ex vivo study showed that certain biomechanical parameters of the CVS were altered after the application of different CXL protocols in porcine eyes. The eyes were measured with strip extensiometry after CVS measurement and showed increased corneal stiffness after treatment.^25^

In particular, the IIR value has been shown to be a clinically relevant parameter, with a lower value after CXL indicating a less deformable cornea.^8^^,^^10^^,^^11^^,^^25^^,^^30^^–^^33^ In contrast, this parameter is higher in KC eyes compared with healthy eyes, indicating a more deformable response to the air puff.^34^ A mean change in IIR between −0.8 and −1.16 mm^−1^ has been reported in several studies and in one previous study even using the same device as in the current study.^9^ The mean changes for IIR in this study were −0.7 ± 0.9 (−1.0 to −0.5), −0.6 ± 1.1 (−0.9 to −0.2), and −0.4 ± 0.7 (−0.7 to −0.1) for the mechanical epi-off CXL group, the tPTK-asst. epi-off CXL group, and the Wf-guided tPRK CXL group, respectively. These changes were more pronounced in the mechanical epi-off CXL group than in the Wf-guided tPRK CXL group, but confirmed the noninferiority of the Wf-guided tPRK CXL group. No statistical significance was found among the groups, but this is of low statistical power, which can be attributed to the study design. Nevertheless, this result is interesting because the cornea became thinner in all groups and therefore a lower resistance to the air puff would be expected as there is a negative relationship between corneal thickness and IIR, that is, the higher the corneal thickness, the lower the IIR, or vice versa, the lower the corneal thickness, the higher the IIR.^34^ On the other hand, corneal thickness after CXL is known to be underestimated by Scheimpflug imaging compared to OCT or ultrasound pachymetry up to 6 months after treatment.^28^^,^^35^^,^^36^ In the Wf-guided tPRK CXL group, however, the cornea actually became thinner as a result of the stromal ablation, but this had only a slight effect on the biomechanical outcome, as the IIR also decreased significantly in this group. This is clearly due to CXL, which increased the overall stiffness of the cornea. Especially after refractive surgery with lasers, such as PRK, LASIK, or SMILE, IIR has been shown to increase significantly due to the distinctive reduction in corneal thickness.^37^ The average removal of the CCT was between 75 and 100 µm for these procedures.^37^

Not many studies have investigated SSI after CXL, however, one of the available studies found that the parameter was increased after accelerated CXL (9 mW/cm² for 10 minutes) with a mean change of +0.08, indicating an increase in corneal material stiffness.^8^ Similarly, in the current study, SSI increased significantly by +0.05 ± 0.12 (0.01 to 0.09), +0.05 ± 0.13 (0.01 to 0.09), and +0.09 ± 0.19 (0.02 to 0.16) for the mechanical epi-off CXL group, the tPTK-ass. epi-off CXL group, and the Wf-guided tPRK CXL group, respectively, with no detectable differences between groups, although the statistical power was low. In another study, no changes in SSI were observed postoperatively at 1 month, 6 months, and 12 months.^9^ No reason for this could be found, as the SSI changed statistically significantly when corneal thickness was included as a covariate in the analysis.^9^ The SSI is a new parameter that describes the material stiffness of the cornea by reducing the effect of corneal thickness and IOP on the parameter.^22^ A recent study has shown that SSI is reduced in KC eyes compared with healthy eyes, indicating less stiffness in these eyes.^30^ Furthermore, the SSI parameter did not change after PRK despite the removal of the corneal tissue, suggesting that it reflects the material stiffness of the cornea, which is less affected by surface laser treatment.^37^ Instead, the reduction in corneal thickness due to stromal ablation has a greater effect on the overall stiffness of the cornea.^37^ In addition, the parameter was developed using numerical modeling, and the type of PRK treatment is more consistent with the assumptions of the model.^37^ In the current study, the material stiffness was increased by CXL and the SSI value increased accordingly, regardless of whether stromal tissue was ablated or not.

Another observation was the increase in bIOP after treatment, which was also seen in our previous studies, with the explanation that these changes may also indicate a stiffer behavior against the air-puff.^9^^,^^25^

The CVS measurement is highly repeatable^38^ and is not influenced by the CXL treatment itself,^8^ so the measured differences between the pre- and postoperative conditions can be considered real clinical changes. The mean percentage change of IIR was −5.9%, −4.8%, and −3.6% for the mechanical epi-off CXL group, the tPTK-ass. epi-off CXL group, and the Wf-guided tPRK CXL group, respectively, and exceeded the previously reported coefficient of variation (preoperative = 3.7% and postoperative = 4.0%).^8^ Similarly, the mean percentage of the SSI was 7.3%, 6.9%, and 9.8% for the mechanical epi-off CXL group, the tPTK-ass. epi-off CXL group, and the Wf-guided tPRK CXL group, respectively, which was higher than the previously reported coefficient of variation (preoperative = 6.2% and postoperative = 6.5%).^8^ The CXL effect outweighs the measurement noise of both parameters. Therefore, the observed clinical changes in the IIR and SSI are not due to decreased reliability of the parameters, but are caused by the CXL treatment and indicate corneal stiffening.

This study also investigated the predictive factors for the change in stiffness-related parameters. Multivariate regression analysis revealed a significant association between pre- and postoperative changes in IIR (Δ IIR) and preoperative IIR, but neither the measured change in TCT (Δ TCT) nor the different groups had an effect on Δ IIR. The higher the preoperative IIR was, the greater was the postoperative decrease in IIR observed in all groups. This was also a conclusion of a previous study by Vinciguerra et al.^11^ and was confirmed by another study that used the Corvis Biomechanical Factor, a system for grading KC, and showed that a higher stage of KC resulted in a greater decrease in IIR.^9^ For SSI, no predictive factor could be found in the analysis.

In addition, the relationship between the amount of laser ablation and the changes in bIOP (Δ bIOP), IIR (Δ IIR), SSI (Δ SSI), CCT (Δ CCT), or TCT (Δ TCT) were also analyzed statistically. The ablation depth of wavefront-guided tPRK combined with CXL affects Δ CCT and Δ TCT, but not the biomechanical parameters. This suggests that a stromal ablation threshold of 50 µm or less might be a safe protocol in terms of postoperative corneal biomechanical stiffening. However, this protocol should be used with caution and possible complications, for example, re-progression after treatment, must be discussed with the patient. Further long-term studies are needed to confirm that the re-progression rate of the wavefront-guided tPRK combined with CXL treatment is not higher than the regular failure rate of CXL (in terms of topographic re-progression).

Tomographic results should be treated with caution because the cornea is still healing. Therefore, visual and tomographic results should be analyzed at earliest 12 months after treatment. However, a typical steepening of K max was observed in the mechanical epi-off CXL group 1 month after treatment, which should disappear after 12 months.^13^ This was not the case for the tPTK-ass. epi-off CXL group and the Wf-guided tPRK CXL group, as stable results or even a flattening effect was observed in the Wf-guided tPRK CXL group after 1 month though. In addition, the ISV, a measure of the deviation of individual corneal radii from the median, and the RMS of anterior high-order aberrations decreased significantly in the Wf-guided tPRK-CXL group. These results suggest that visual acuity will improve in these patients with longer follow-up.

The study is limited by its retrospective and non-randomized design. Furthermore, wavefront guided ablation patterns were generated from anterior or total cornea measurements, which might influence the stromal ablation depth as anterior corneal aberrations are higher than total cornea aberrations.^39^ Because there was no aberrometry device available in the clinical setting, laser ablation patterns based on total ocular aberrations have not been considered. Future studies could also investigate the longitudinal elastic modulus using the Brillouin microscopy^40^ after Wf-guided tPRK-CXL, which would provide depth-dependent biomechanical properties. Visual outcomes could not be determined at the time of this analysis because these only provide reliable results at 1 year postoperatively. This can also be addressed with long-term data.

In conclusion, the biomechanical changes of the cornea induced by epi-off CXL are independent of the method of epithelial ablation. In particular, wavefront-guided tPRK in combination with CXL resulted in a stiffening effect of the keratoconic cornea despite the removal of stromal tissue. This observation applies to treatments with an estimated stromal ablation of no more than 50 µm in the cone area. The early biomechanical results suggest that wavefront-guided tPRK in combination with CXL appears to be a safe treatment for keratoconus as tomographic improvements were also observed, possibly leading to an improvement in visual acuity in long-term follow-up.
